# Prevalence of Hearing Loss in Newborns Admitted to Neonatal Intensive Care Unit

**Published:** 2012

**Authors:** Shahnaz Pourarian, Bijan Khademi, Narjes Pishva, Ali Jamali

**Affiliations:** 1 *Neonatal Research Center, Faculty of medicine, Shiraz University of Medical Sciences, Shiraz, Iran*; 2 *Department of otorhinolaryngology, Khalili Hospital, Faculty l of medicine, Shiraz University of Medical Sciences, Shiraz, Iran*

**Keywords:** Auditory brainstem response, Hearing loss, Newborn, Neonatal intensive care unit, Otoacoustic emission

## Abstract

**Introduction:**

Hearing is essential for humans to communicate with one another. Early diagnosis of hearing loss and intervention in neonates and infants can reduce developmental problems. The aim of the present study was to assess the prevalence of hearing impairment in newborns admitted to a neonatal intensive care unit (NICU) and analyze the associated risk factors.

**Materials and Methods:**

This cross-sectional study was conducted to assess the prevalence of hearing loss in neonates who were admitted to the NICU at Nemazee Hospital, Shiraz University of Medical Sciences between January 2006 and January 2007. Auditory function was examined using otoacoustic emission (OAE) followed by auditory brainstem response (ABR) tests. Relevant potential risk factors were considered and neonates with a family history of hearing loss and craniofacial abnormality were excluded. For statistical analysis logistic regression, the chi-squared test, and Fisher’s exact test were used.

**Results:**

Among the 124 neonates included in the study, 17 (13.7%) showed hearing loss in the short term. There was a significant statistical relationship between gestational age of less than 36 weeks (P=0.013), antibiotic therapy (P= 0.033), oxygen therapy (P=0.04), and hearing loss. On the contrary, there was no significant relationship between hearing loss and use of a ventilator, or the presence of sepsis, hyperbilirubinemia, congenial heart disease, transient tachypnea of newborn, congenital pneumonia, or respiratory distress syndrome.

**Conclusion:**

Auditory function in neonates who are admitted to a NICU, especially those treated with oxygen or antibiotics and those born prematurely, should be assessed during their stay in hospital. The importance of early diagnosis of hearing loss and intervention in these neonates and avoidance of any unnecessary oxygen or antibiotic therapy needs to be further promoted.

## Introduction

Recent advances in neonatal medicine have increased the survival rate of newborns, especially those admitted to neonatal intensive care units (NICUs). Due to problems such as prematurity, low Apqar scores, infection, and hyperbilirubinemia, and the risks associated with treatment strategies including mechanical ventilation, oxygen therapy, administration of antibiotics and other medications, infants in NICUs face various problems including hearing impairment. Significant hearing loss is the most common disorder at birth, occurring in 1 to 2 newborns per 1000 in the general population and 24% to 46% (1,2) of newborns who are admitted to a NICU. The incidence of hearing impairment in neonates in Iran has been shown to 8% in high-risk neonates and 16% in neonates in intensive care (3). 

Many factors might play a role in placing these NICU babies at an increased risk of hearing loss, including underlying disease processes as well as the treatment they receive (4,5). In addition to periods of profound hypoxia-ischemia, infants with respiratory failure may be treated with hyperventilation or alkalizing therapy, which might compromise the oxygenation and perfusion of the cochlear organ and auditory pathway, resulting in hearing loss (6,7). The use of ototoxic drugs, including loop diuretics (8) and aminoglycosides, has been associated with increased vulnerability of the cochlear to damage from preexisting hypoxia. Although hearing loss has been described in children who were critically ill as newborns (9), most of the children had received aminoglycosides, diuretics and neuromuscular blocking agents. 

Screening procedures to detect hearing impairment may be divided into two categories: behavioral and electrophysiological. Behavioral techniques produce a high number of false-negative results due to the relative subjectivity of the assessment and difficulty in detecting mild or unilateral hearing loss. Electrophysiological procedures have greater sensitivity and specificity and include measuring the following: auditory brainstem responses (ABR), automated auditory brainstem responses (AABR), and evoked otoacoustic emissions (EOAE) (10). Various studies have analyzed the cost of auditory screening in the neonatal period as well as the differences between the methods available (11). The objective of the present study was to assess the prevalence of hearing impairment using measurements of ABR and OAE in newborns admitted to a NICU.

## Materials and Methods

 An observational cross-sectional study was conducted between January 2006 and January 2007. All the newborns admitted to the NICU at Nemazee Hospital, which is affiliated to Shiraz University of Medical Sciences, were included in the study. Information about the condition of each neonate was collected in the form of a questionnaire and included: gestational age (New Ballard method); family history of congenital hearing loss and consanguinity; presence of conditions including asphyxia (Apqar score < 4), sepsis, respiratory distress syndrome, transient tachypnea of newborn (TTN), congenital pneumonia, congenial heart disease (CHD), or hyperbilirubinemia (20 mg/d1); and treatments used including phototherapy (>2 days), mechanical ventilation (>5 days), antibiotic therapy including aminoglycosides (>5 days), or oxygen therapy (>1 week and > 40% FIO2). Babies who had craniofacial malformation and those with a family history of deafness were excluded from the study. 

The presence of unilateral or bilateral hearing loss was considered as deafness in this study. At the time of discharge from the hospital, infants were tested using OAE and ABR screening. Infants were screened by using distortion-product OAEs (DPOAEs), set at a 2 to 5 kHz screen with 3 of 4 frequency bands being required to be present for a pass. The intensity was calibrated at a 65 dB sound pressure level for band 1 and 55 dB sound pressure level for band 2. Parameters for the ABR included a 100 μs click stimulus, alternating polarity, a click rate of 37.1 clicks per second, and an intensity of 35 dB. The low-pass filter was set at 1500 Hz, and the high pass filter was set to 100 Hz; a minimum of 1536 sweeps up to a maximum of 2 trials of 6144 sweeps were obtained.

A patented signal-detection algorithm, the point-optimized variance ratio, determined the presence of an ABR and assigned a pass or fail result. A point-optimized variance ratio score of 3.50 was necessary for a pass. The specificity of the screening ABR test is in excess of 98.5%. The verified theoretical sensitivity for the ABR test is 99.96% (G. Raviv, PhD, Bio-logic Corp, written communication, 2005). The result was considered normal when the newborn responded to a 35 dB signal in both ears separately and impaired when a response to 35 dB was not detected in at least one ear. The data were analyzed by an ENT specialist. In this study logistic regression, the chi-squared test and Fisher’s exact test were used and P-values of less than 0.05 were accepted as significant. The protocol was assessed and approved by the Research Ethics Committee of the Medical Council of I.R.I. and informed consent was obtained from the parents. 

## Results

A total of 124 neonates were included in this cross-sectional study and 17 of these had hearing loss. Statistical analyses in the form of logistic regression were performed to assess the risk factors associated with hearing loss. The relationship of oxygen therapy with hearing loss was analyzed using the chi-squared test and in some cases Fisher’s exact test was used due to low sample numbers. 

**Fig 1:**
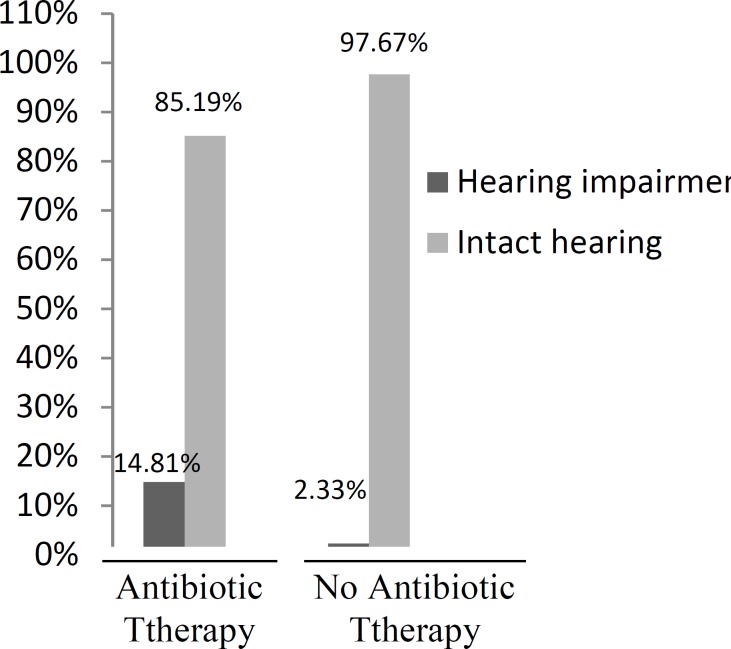
Relationship between antibiotic therapy and hearing loss

Out of the total study group, 11 (13.6%) of the 73 boys and 6 (11.7%) of the 51 girls showed evidence of hearing loss. Statistical analysis indicated that hearing loss was not related to gender (P=0.95). Of the 69 neonates who were born at a gestational age of greater than 36 weeks, 4 (2.42%) had sensorineural hearing loss. In contrast, 10 (8.06%) of the 55 preterm neonates (less than 36 weeks) had hearing loss and there was a statistically significant relationship between gestational age and hearing loss (P=0.013). A total of 7 (14.8%) of the 47 neonates with hyperbilirubinemia had bilirubin levels of greater than 20 mg/dl and required exchange transfusion but only 2 (28.5%) of them had hearing loss. There was no statistically significant relationship between hearing loss and bilirubin levels of greater than 20 mg/dl. In terms of medical treatments, of the 105 cases who were administered antibiotics (aminoglycoside) 19 (14.8%) had hearing loss, which was a significantly greater number than that observed in the babies who did not receive antibiotics (P=0.00; Fig. 1). 

**Fig 2:**
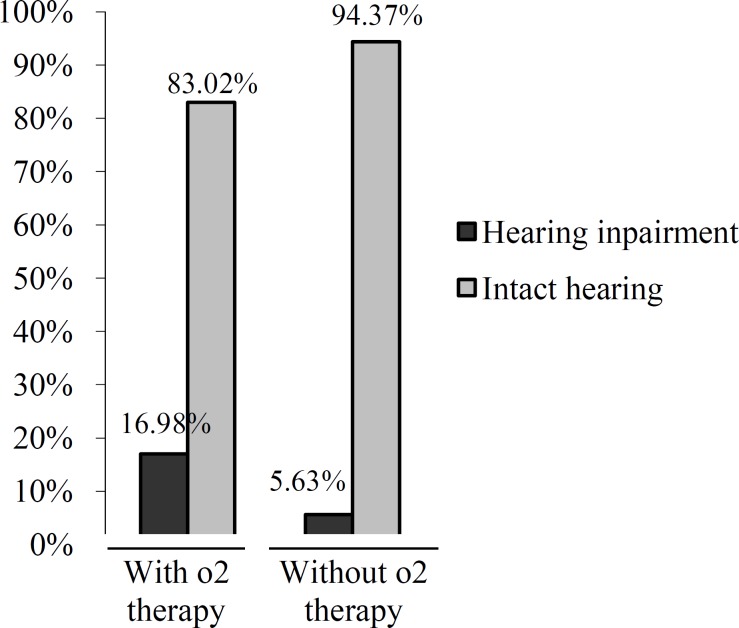
Relationship between oxygen therapy and hearing loss

For oxygen therapy, 21 (17.0%) of the 103 babies who received the therapy (>1 week and >40% FIO2) also had hearing loss, which was a statistically significant result (chi-squared (1, 124) = 4.16, P=0.041; Fig. 2).

There was no statistically significant relationship between hearing loss and the other risk factors identifed including respiratory distress syndrome (28%), TTN (4.8%), congenital pneumonia (6.4%), CHD (3.2%), mechanical ventilation for longer than 5 days (8.8%), hyperbilirubinemia of less than or equal to 20 mg/dl (5.6%), phototherapy for more than 2 days (20%), asphyxia (Apqar score < 4 [6.4%]), and sepsis (32%). The average duration of hospitalization was 21 days (range: 12–36 days).

## Discussion

It is generally accepted that screening for hearing loss in neonates is crucial, and it has been reported that the use of a universal newborn hearing screening is more valuable than screening just those who have been admitted to a NICU (12). Recognizing and treating hearing loss in its early phase is of critical value, but the economic aspects of screening should be considered (11).

In this study on 124 neonates admitted to the NICU of Nemazee Hospital, Shiraz University of Medical Sciences, hearing loss was confirmed in 17 cases indicating the importance of screening for hearing loss in this category of neonates. In order to determine the relative risk factors associated with hearing loss in neonates we considered only those who had be admitted to the NICU as the incidence of hearing impairment is 24% to 46% in this group. Infants were tested using OAE and ABR, which has been suggested by previous reports to be a successful two-step screening protocol for at risk babies (13,14).

Our results indicate that as the prevalence of hearing loss in newborns with a gestational age of less than 36 weeks was clearly higher, prematurity should be considered as a risk factor for hearing loss in the NICU population. These babies face problems due to the underdevelopment of their respiratory system, which requires more and longer oxygen therapy, and as a consequence of their weakened immune system they are also exposed to various infections and thus the chance of sepsis and the use of antibiotic therapy will be higher. Both of these variables are significantly associated with hearing loss in our study.

It has been reported in literature that adequate oxygenation and perfusion are essential for cochlear function and that hypoxia can lead to hearing loss (15). Our analysis showed a significant relationship between prolonged oxygen therapy and hearing loss. It seems that inappropriate and prolonged oxygen therapy could have negative effects on an infant's hearing system and it is better to refuse undue administration for neonates. Whenever oxygen therapy is necessary, auditory function needs to be monitored during and after this therapy.

The literature considers the use of ototoxic medication to be an important risk factor for deafness in the neonatal period (16,17) and in the present case series, a significant relationship was observed. It should be noted that the drug of choice for the treatment of gram negative organisms at our institution is amikacin, and we believe that strict control of the serum dose of this drug is an important means for preventing hearing loss due to ototoxicity. It may be speculated that improvement in the treatment protocols in NICUs may reduce the probability of auditory involvement.

With regards to mechanical ventilation for more than five days, the data in the literature point to the procedure as an important cause of deafness. In the present study, mechanical ventilation was not identified to be significantly associated with hearing loss, perhaps due to the short duration of ventilation that was used in most cases. The use of phototherapy has also been studied in the literature and we included it as a variable in this analysis because it is a very common procedure in our unit and it involves a high noise level (mean of 45 dB during the day and 35 dB at night, the maximum limit suggested being 58 dB) (18). 

The number of children submitted to phototherapy was higher than the number considered as having hyperbilirubinemia, since the indication for phototherapy includes other children in addition to those with total bilirubin levels of greater than 20 mg/dl. We did not observe an association between the use of phototherapy and hearing impairment. In addition, although severe hyperbilirubinemia is a neonatal risk factor that has been proven to be associated with hearing loss, no significant relationship was observed in the present study. This may be because most of the newborns in the NICU has undergone exchange transfusion before the fatal complications of high bilirubin could occur.

## Conclusion

In conclusion we would like to stress the importance of identifying infants with hearing disorders, administering early treatment, and performing appropriate tests in infants at high risk of hearing impairment. When dealing with these babies treatments such as unnecessary oxygen or antibiotic therapy should be refused.
